# Development and evaluation of a Zr-MOF/PVA nanofiber composite for efficient adsorption of malathion and carbofuran and antimicrobial control of aquatic pathogens

**DOI:** 10.1039/d6ra01485b

**Published:** 2026-05-15

**Authors:** Farag M. A. Altalbawy, Ali Abdulrahman Alsalamy, Shahad Mohammed Dhiaa Younis, Karar H. Alfarttoosi, Waam Mohammed Taher, Mariem Alwan, Mahmood Jawad, Hiba Mushtaq, Irfan Ahmad, Aseel Smerat

**Affiliations:** a Department of Chemistry, University College of Duba, University of Tabuk Tabuk Saudi Arabia; b Department of Chemistry, Faculty of Science, Al-Baath University Homs Syria alsalamyali06@gmail.com; c College of Pharmacy, Alnoor University Nineveh Iraq; d College of Pharmacy, Ahl al bayt University Kerbala Iraq; e College of Nursing, National University of Science and Technology Dhi Qar Iraq; f Pharmacy College, Al-Farahidi University Baghdad Iraq; g Department of Pharmacy, Al-Zahrawi University College Karbala Iraq; h Gilgamesh Ahliya University Baghdad Iraq; i Department of Clinical Laboratory Sciences, College of Applied Medical Sciences, King Khalid University Abha Saudi Arabia; j Hourani Center for Applied Scientific Research, Al-Ahliyya Amman University Amman 19328 Jordan

## Abstract

Carbofuran and Malathion are widely used pesticides that pose serious environmental and health risks due to their persistence and high toxicity. In this study, a novel Zr/H_3_Imdc/PVA nanofibrous composite was synthesized *via* microwave-assisted MOF formation followed by electrospinning and evaluated for simultaneous pesticide adsorption and antimicrobial activity. Characterization confirmed successful composite formation, with a high specific surface area and uniform nanofibrous morphology enriched with oxygen- and nitrogen-containing functional groups. The Zr/H_3_Imdc-MOF exhibited a high specific surface area of 1560 m^2^ g^−1^, which increased to 1755 m^2^ g^−1^ upon incorporation into the PVA nanofibrous matrix, indicating preserved porosity and enhanced accessibility of active sites. Adsorption experiments showed maximum removal efficiencies of 94.9% for Malathion and 91% for Carbofuran using 0.04 g L^−1^ adsorbent at neutral pH and 50 °C, with optimal contact times of 90 and 120 minutes, respectively, for an initial concentration of 400 mg L^−1^. The high adsorption is attributed to the combined effect of Zr active sites, imidazole functional groups, and the nanofibrous PVA structure. Additionally, the composite exhibited notable antibacterial activity against five pathogenic aquatic bacterial strains, likely due to zirconium species, imidazole moieties, and enhanced surface interactions. These results demonstrate that the Zr/H_3_Imdc/PVA nanofiber composite is a multifunctional material with promising potential for water purification, providing both pesticide removal and microbial control.

## Abstract 2

Zr/H_3_Imdc/PVA nanofiber composite were developed for dual-function wastewater treatment: efficient pesticide removal and potent antibacterial activity against key aquatic pathogens, addressing a critical gap in aquaculture health management.

## Graphical abstract

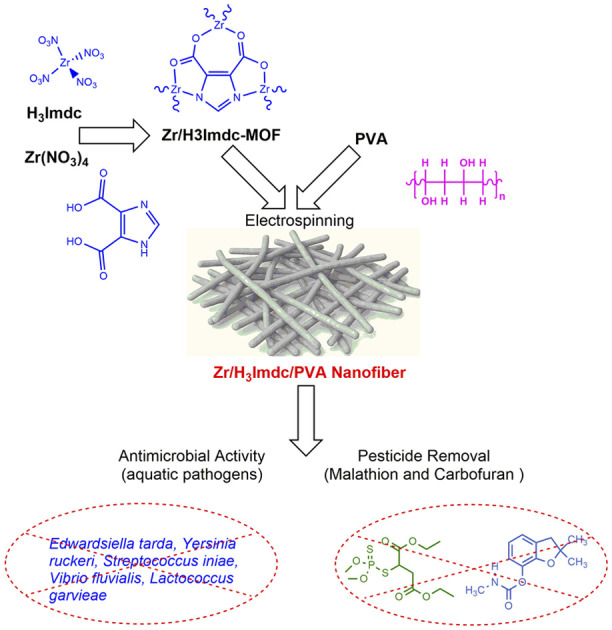

## 1 Introduction

Water contamination by emerging chemical and biological pollutants has become one of the most critical global environmental challenges in recent decades. Agricultural activities, industrial effluents, and improper waste management have led to the continuous release of hazardous compounds into aquatic environments, threatening ecosystems and public health. Conventional water treatment technologies often suffer from limitations such as low removal efficiency, high operational costs, secondary pollution, and limited effectiveness against complex mixtures of contaminants.^1,2^

In this context, advanced materials based on nanotechnology have attracted increasing attention due to their high surface area, tunable surface chemistry, and multifunctional capabilities. Among these materials, metal–organic frameworks (MOFs) and MOF-based nanocomposites have demonstrated remarkable potential for water purification applications, particularly for the adsorption of toxic organic pollutants and the inhibition of pathogenic microorganisms. The integration of MOFs with polymeric matrices, such as polyvinyl alcohol (PVA), offers additional advantages including improved mechanical stability, processability, and enhanced interaction with target contaminants.^3,4^

Zirconium-based MOFs have attracted considerable attention due to their high chemical stability, strong metal–ligand bonding, and abundant surface-active sites, which make them suitable for aqueous-phase adsorption processes. The use of 1*H*-imidazole-4,5-dicarboxylic acid (H_3_Imdc) as an organic linker introduces nitrogen- and oxygen-containing functional groups that can enhance interactions with pesticide molecules through hydrogen bonding and coordination mechanisms. Furthermore, incorporation of the MOF into a polyvinyl alcohol (PVA) nanofibrous matrix improves mechanical strength, prevents particle agglomeration, and increases the accessibility of active sites by providing a high-surface-area, processable structure.^3,5^

Despite extensive research on nanomaterial-based adsorbents for pesticide removal, several critical challenges remain unresolved. Numerous MOF-based materials demonstrate significant adsorption capacity yet are hindered by inadequate mechanical stability, particle agglomeration, or a singular focus on the removal of chemical pollutants. In addition, most studies do not address the simultaneous presence of chemical contaminants and pathogenic microorganisms in water systems. These limitations highlight the need for robust, multifunctional materials capable of efficient pesticide adsorption while providing antimicrobial activity under practical operating conditions.^6^

Among various water pollutants, agricultural pesticides represent a major class of contaminants due to their extensive use and high potential for environmental dispersion.

**Fig. 1:**
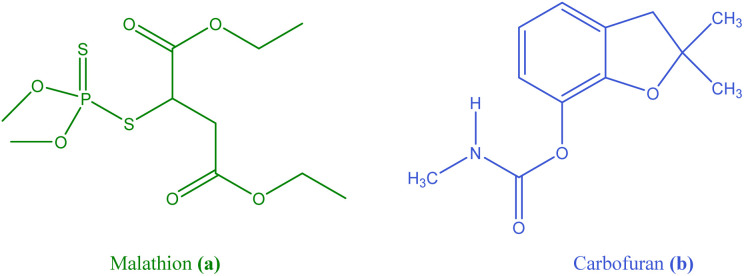
Structure of Malathion (a) and Carbofuran (b).

Malathion (diethyl 2-((dimethoxyphosphorothioyl)thio)succinate/Fig. 1a) is an organophosphate insecticide widely used for pest control in agricultural and residential settings, and it is typically a colorless to yellow-brown liquid with a distinctively unpleasant odor.^7,8^ Malathion is widely utilized for agricultural pest control (effective against various pests affecting crops),^9^ public health (used in mosquito control programs and for treating lice infestations), and residential areas (commonly applied in gardens and landscaping to manage household insect populations).^10^ While Malathion is considered less toxic to mammals than other organophosphates, it can still cause adverse health effects in humans, including weakness, abdominal pain, nausea and vomiting, and blurred vision.^11^ Malathion is moderately soluble in water and can contaminate surface waters and groundwater. The half-life of Malathion ranges from 2 to 18 days, influenced by various environmental factors such as temperature, pH, and moisture levels.^12^ The compound poses significant risks to non-target organisms, particularly beneficial insects like honeybees and aquatic life. It is highly toxic to bees and some fish species while exhibiting moderate toxicity to birds.^13^

Carbofuran (2,2-dimethyl-2,3-dihydrobenzofuran-7-yl methylcarbamate/Fig. 1b), marketed under trade names such as Furadan and Curaterr 10 GR, is a bicyclic organic compound that consists of benzene and furan linked together, featuring a methylcarbamate group. This compound is primarily used in the agricultural industry for pest control, particularly in cultivating crops such as vegetables and eggplant.^14^ Carbofuran is highly toxic and can lead to hormonal and reproductive changes through endocrine disruption.^15^ Exposure to Carbofuran can cause a range of symptoms including weakness, abdominal pain, nausea and vomiting, loss of coordination and balance, muscle contractions or twitching, blurred vision, and other cholinergic effects such as miosis (pupil constriction) and increased salivation.^16^ This toxic chemical compound poses significant risks not only to humans but also to a wide range of other organisms, including birds, aquatic animals, and terrestrial wildlife.^17^ Due to its solubility in water, its entry into water sources and groundwater can lead to pollution.^18^ Due to its chemical properties, Carbofuran has a high potential for leaching into groundwater.^19^ While it is not persistent in soil, it may persist in water under certain conditions, making it hazardous to aquatic ecosystems.^20^ The compound is highly toxic to birds and honeybees, exhibiting moderate to high toxicity to most marine organisms.^21^

In real water environments, chemical contamination by pesticides is often accompanied by microbial pollution, particularly pathogenic bacteria that threaten aquatic organisms and human health. Therefore, developing multifunctional materials capable of simultaneously removing chemical pollutants and inhibiting microbial growth is of significant practical importance. Materials containing metal ions and heterocyclic ligands, such as zirconium and imidazole derivatives, have shown potential antimicrobial activity, making them attractive candidates for dual-function water treatment applications.^3^

In addition to chemical contaminants, certain bacterial strains present in water can cause diseases in aquatic animals that may be transmitted to humans through consumption. Among these bacteria are *Edwardsiella tarda*, *Yersinia ruckeri*, *Streptococcus iniae*, and *Lactococcus garvieae*, which in some cases can cause illness and even death in humans.^22–24^

*Edwardsiella tarda* is a common pathogen in freshwater and marine aquaculture worldwide,^25^*Yersinia ruckeri* is the causative agent of enteric redmouth disease in salmonids,^26^*Streptococcus iniae* is a major concern in tilapia and hybrid striped bass farming,^27^ and *Lactococcus garvieae* is an emerging pathogen in European and Asian aquaculture.^28^

Due to the antibiotic resistance exhibited by certain bacterial strains, the use of new technologies, especially nanotechnology, has become increasingly important in combating resistant bacteria. To date, many nanocompounds have been synthesized and developed that possess antimicrobial properties against Gram-positive, Gram-negative, and specialized aquatic bacterial strains.^29^

The removal of Carbofuran and Malathion using nanotechnology has gained significant attention due to the effectiveness of various nanomaterials, including carbon nanotubes (CNTs),^30^ metal oxide nanoparticles, and nanocomposites,^31,32^ nanographene oxide,^33,34^ nanofibers,^35,36^ and metal–organic frameworks (MOFs).^37,38^

The d block metals such as copper (Cu), iron (Fe), zinc (Zn), zirconium (Zr) and cobalt (Co) have been widely used to synthesize MOF based nanofiber composites for various applications. For example, Cu MOF/PVA nanofibers have shown promising antibacterial activity,^39^ while Fe MOF/PAN nanofibers have been explored for organic dye removal.^40^ Zinc based MOF nanofibers have also been investigated for energy storage applications.^41^ However, the comparative performance of these alternative metal based composites for simultaneous pesticide adsorption and antimicrobial activity against aquatic pathogens remains to be investigated. The present study focuses on zirconium due to its high chemical stability, strong metal–ligand bonding, and abundant surface active sites, which make it particularly suitable for aqueous phase adsorption processes.

Metal–organic frameworks (MOFs) synthesized from Zr have shown great potential in removing organic pollutants.^42^ Their high porosity and tunable structure allow them to capture pesticides efficiently. The incorporation of MOFs into nanofibers enhances the overall adsorption performance.^43^

The synthesis of MOF/polymer nanofiber composites *via* electrospinning has gained significant attention in recent years. Several comprehensive reviews have summarized the progress in this field, covering fabrication strategies, material design, and applications in environmental remediation, energy storage, and biomedicine.^5,44^ Specifically, the integration of MOFs into electrospun nanofibers has been shown to improve mechanical stability, prevent particle agglomeration, and increase the accessibility of active sites.^45–47^ The present study builds upon these advances by applying the electrospinning technique to fabricate a Zr/H_3_Imdc/PVA composite tailored for removal efficiency of hazardous pesticides such as Carbofuran and Malathion.

In this hybrid system, polyvinyl alcohol (PVA) plays a crucial role beyond serving as a supporting polymeric matrix. The abundant hydroxyl (–OH) groups of PVA can form hydrogen bonds with both the Zr/H_3_Imdc-MOF and pesticide molecules, facilitating improved interfacial interactions. Moreover, the incorporation of PVA enhances the mechanical stability and dispersion of MOF particles within the nanofibrous structure, preventing agglomeration and increasing the accessibility of active adsorption sites. These combined effects contribute to improved adsorption efficiency and structural integrity of the composite material.^3^

In general, the performance of adsorbent materials is often evaluated based on parameters such as contact time, pH, initial concentration of pesticides, and temperature. Based on these considerations and our experimental findings, the synthesized Zr/H_3_Imdc/PVA composite demonstrates high potential for the efficient removal of hazardous pesticides that pose serious risks to humans, aquatic organisms, and beneficial insects. Accordingly, this study focuses not only on the removal of chemical contaminants from water but also on the control of microbial pollutants, including pathogenic bacterial strains affecting aquatic organisms, such as *Edwardsiella tarda*, *Yersinia ruckeri*, *Streptococcus iniae*, *Vibrio fluvialis*, and *Lactococcus garvieae*. The antimicrobial properties of the synthesized Zr/H_3_Imdc/PVA against these strains were also investigated.

The novelty of the present work lies in three key aspects. First, while Zr-MOF/PVA composites have been reported previously, the specific combination of microwave-assisted synthesis of Zr/H_3_Imdc-MOF followed by electrospinning into PVA nanofibers has not been explored for pesticide adsorption. Second, this study evaluates the composite for simultaneous removal of two structurally different pesticides (Malathion and Carbofuran), whereas most previous reports focus on single contaminants. Third, to the best of our knowledge, this is the first report on the antimicrobial activity of a Zr/H_3_Imdc/PVA nanofiber composite against five specific aquatic pathogens (*Edwardsiella tarda*, *Yersinia ruckeri*, *Streptococcus iniae*, *Vibrio fluvialis*, and *Lactococcus garvieae*), which are relevant to both aquaculture and human health.

Accordingly, the objectives of this study are to: (i) synthesize a novel Zr/H_3_Imdc/PVA nanofibrous composite with high structural stability and surface activity; (ii) evaluate its adsorption performance toward Malathion and Carbofuran under various operational conditions, targeting removal efficiencies above 90% at low adsorbent dosages (<0.05 g L^−1^); and (iii) investigate its antimicrobial activity against selected pathogenic bacterial strains relevant to aquatic environments. This integrated approach aims to address both chemical and biological water contamination using a single multifunctional material.

## 2 Experimental

### 2.1 Material

The 1*H*-imidazole-4,5-dicarboxylic acid (H_3_Imdc) with 98% purity and Zr(NO_3_)_4_ with 98% purity were obtained from TCI, and Merck company. Polyvinyl alcohol, Mw 9000–10 000, 80% hydrolyzed were obtained from Merck. The Malathion and Carbofuran stock standard solutions were purchased from Sigma-Aldrich.

The BP211 microwave and Inovenso NE100 were used to synthesis of Zr-MOF and nanofiber. The XRD, FT-IR, CHNO Elemental analysis, BET, and SEM were prepared using Shimadzu XRD 7000, PerkinElmer RX1 FT-IR spectrometer, LECO TruSpec Elemental analysis, S4300 TOB BET surface area analyzer, S-3200 Hitachi SEM.

The PerkinElmer Clarus 680, Gas Chromatograph (Electron Capture Detector) and PerkinElmer Clarus SQ 8C Mass Spectrometry were used to determination of Malathion adsorption. The Agilent 1200, UV-detector and C18 column were used to determination of Carbofuran adsorption.

### 2.2 Synthesis of Zr/H_3_Imdc/PVA

For synthesis Zr/H_3_Imdc/PVA, 1 mmol of 1*H*-imidazole-4,5-dicarboxylic acid (H_3_Imdc) and 1.5 mmol of Zr(NO_3_)_4_ were added to 20 mL of deionized water and stirred to obtain a homogeneous mixture. This mixture was then subjected to microwave radiation at 350 W for 25 min. Following this step, the product underwent nanofiltration separation. It was then washed with a water/ethanol (1 : 1) solution and subsequently dried under vacuum in an oven at 100 °C for 4 hours. This process resulted in the successful formation of Zr/H_3_Imdc-MOF.^48^

Electrospinning was carried out using a standard single-needle setup. The polymer solution was fed through a metallic needle under a high-voltage electric field, and the fibers were collected on a grounded collector at room temperature under controlled ambient conditions. So, 0.01 mg of the Zr/H_3_Imdc-MOF was dispersed in 20 mL of deionized water, and 20 mL of a 0.004% solution of polyvinyl alcohol (PVA) in acetic acid was added. The resulting mixture was stirred for 20 min at room temperature. After homogenization, electrospinning was performed at a voltage of 28 kV, with a flow rate of 0.4 mL h^−1^ and a needle-to-collector distance of 22 cm. Finally, after the solvent evaporated, the Zr/H_3_Imdc/PVA was synthesized.^39,49^

### 2.3 Adsorption of malathion using Zr/H_3_Imdc/PVA

In the study of Malathion adsorption, concentrations of 1–100 mg L^−1^ were prepared and tested with the dosage of 0.01 g L^−1^ to 0.1 g L^−1^of Zr/H_3_Imdc/PVA at pH 4–11 by HCl (0.1 M) and NaOH (0.1 M) solutions, temperatures of 25 °C to 60 °C and 30 min to 180 min. Following centrifugation and separation of the Zr/H_3_Imdc/PVA, the residual solution was analyzed to determine the concentration of Malathion using gas chromatography coupled with an electron capture detector.

Gas chromatographic analysis was carried out using a capillary column suitable for pesticide analysis, with injector and detector temperatures. The oven temperature program was optimized to achieve adequate separation of Malathion [column type (HP-5 capillary column, 30 m × 0.32 mm × 0.25 µm), injector temperature (250 °C), detector temperature (300 °C), oven temperature program (initial 150 °C for 2 min, ramped to 280 °C at 10 °C min^−1^), carrier gas (nitrogen at 1.2 mL min^−1^), and injection volume (1 µL)].

Quantitative analysis of Malathion was carried out using an external calibration method based on standard solutions with different concentrations. Calibration curves showed acceptable linearity (*R*^2^ > 0.99). All measurements were performed under identical chromatographic conditions to ensure reproducibility.

The adsorption percentage or *R*_e_ (%) was obtained using eqn (1).^50^

1 *R*_e_ (%) = [(*C*_0_ − *C*_e_)/*C*_0_] ×100

*C*_0_: initial concentration of Malathion (mg L^−1^). *C*_e_: equilibrium concentration of Malathion (mg L^−1^).

Eqn (1). Calculation of the adsorption percentage of Malathion.

### 2.4 Adsorption of carbofuran using Zr/H_3_Imdc/PVA

In the study of Carbofuran adsorption, concentrations of 1–100 mg L^−1^ were prepared and tested with the dosage of 0.01 g L^−1^ to 0.1 g L^−1^ of Zr/H_3_Imdc/PVA at pH 4–11 by HCl (0.1 M) and NaOH (0.1 M) solutions, temperatures of 25 °C to 60 °C and 30 min to 180 min. The experiments were conducted using 50 mL of pesticide solution under constant agitation at 200 rpm. Finally, after centrifugation and separation of Zr/H_3_Imdc/PVA, the amount of Malathion in the remaining solution was measured by High-Performance Liquid Chromatography (HPLC). The HPLC analysis was performed using a C18 reversed-phase column with UV detection at an appropriate wavelength. The mobile phase consisted of a mixture of water and organic solvent, delivered at a constant flow rate, and the injection volume was kept constant for all samples [column (C18, 250 mm × 4.6 mm, 5 µm), mobile phase (acetonitrile : water 60 : 40 v/v), isocratic elution, flow rate (1.0 mL min^−1^), column temperature (25 °C), injection volume (20 µL), detection wavelength (220 nm for Carbofuran), and run time (15 min)].

Quantification was carried out using external calibration curves prepared from standard Carbofuran solutions. The adsorption percentage or *R*_e_ (%) was obtained using eqn (2).^51^

2 *R*_e_ (%) = [(*C*_0_ − *C*_e_)/*C*_0_] ×100

*C*_0_: initial concentration of Carbofuran (mg L^−1^). *C*_e_: equilibrium concentration of Carbofuran (mg L^−1^).

Eqn (2). Calculation of the adsorption percentage of Carbofuran.

### 2.5 Antibacterial activity of Zr/H_3_Imdc/PVA

The antibacterial activity of the synthesized Zr/H_3_Imdc/PVA (at concentrations ranging from 1 to 1024 µg mL^−1^) was evaluated following CLSI guidelines using standard microbiological assays. The minimum inhibitory concentration (MIC) and minimum bactericidal concentration (MBC) were determined using the broth microdilution method in Mueller–Hinton broth, with incubation at 37 °C for 24 h. The inhibition zone diameter (IZD) was assessed using the disk diffusion method on Mueller–Hinton agar plates.^52,53^ All antimicrobial assays were performed in triplicate, and the reported MIC, MBC, and inhibition zone values represent average results. The experimental variability was within an acceptable range, with standard deviations below 10%. Blank experiments without adsorbent and control tests without antibacterial agents were conducted under identical conditions for comparison. All antimicrobial assays were performed in triplicate, and the results are reported as mean ± standard deviation (SD).

## 3 Results and discussion

### 3.1 Synthesis and structural characterization

Material characterization plays a critical role in understanding the adsorption and antimicrobial performance of the synthesized Zr/H_3_Imdc/PVA composite. Structural, morphological, and surface analyses were therefore correlated with the observed functional properties to elucidate how the material design contributes to its performance in water treatment applications.

#### 3.1.1 Zr/H_3_Imdc/PVA synthesis

The synthesis of Zr/H_3_Imdc-MOF involved using zirconium(iv) nitrate (Zr(NO_3_)_4_) as the metal source and 1*H*-imidazole-4,5-dicarboxylic acid as the organic ligand. This combination allowed for the formation of a stable MOF structure. The reaction was carried out using microwave irradiation, which is discussed in the next section for its role in imparting unique physical and chemical properties to the final product. A power setting of 350 W was employed for the synthesis, based on previous studies that reported these conditions as optimal for achieving acceptable physical and chemical properties in similar MOF products.^48^

**Scheme 1:**
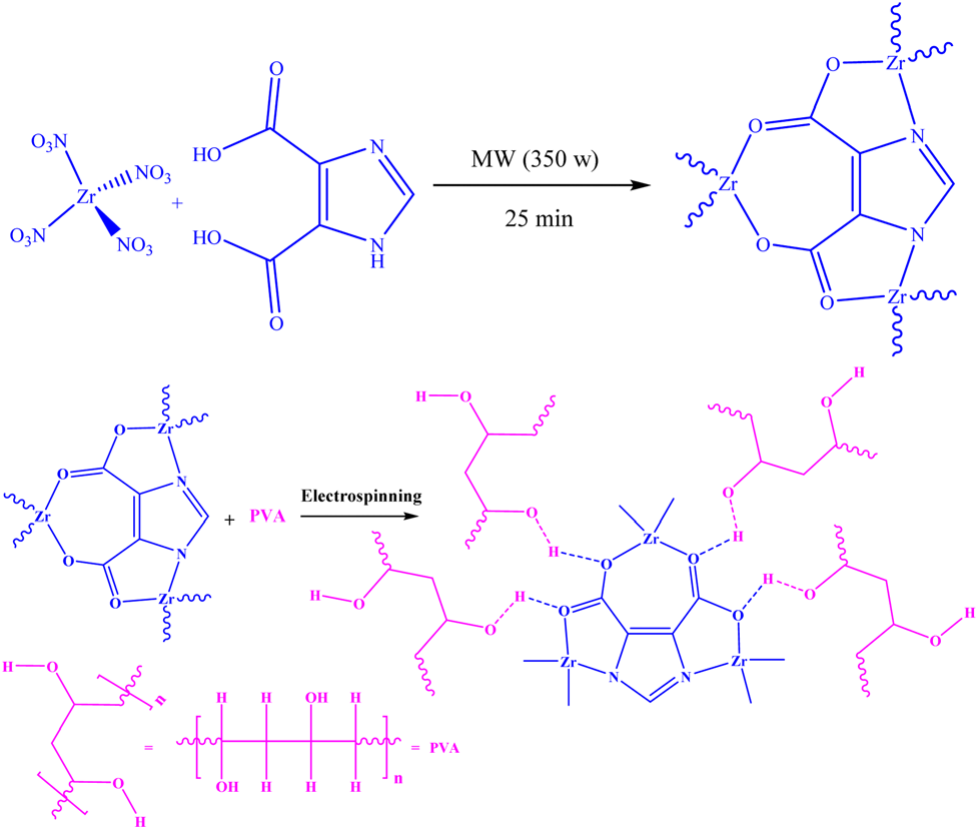
Synthesis of (a) Zr/H_3_Imdc-MOF, and (b) Zr/H_3_Imdc/PVA.

The proposed reaction mechanism for synthesizing Zr/H_3_Imdc-MOF is illustrated in Scheme 1a.

Using the electrospinning method under the optimal conditions reported in previous studies, nanofibers containing Zr/H_3_Imdc-MOF were synthesized. Polyvinyl alcohol (PVA) was utilized as the primary matrix material in the fabrication of nanofibers, providing structural support and facilitating their formation.

According to Scheme 1b, the interaction between the Zr/H_3_Imdc-MOF and PVA occurs through hydrogen bonding between the hydrogen atoms of PVA and the oxygen groups in the MOF.

#### 3.1.2 Characterization

Most of the characterization techniques were common for both Zr/H_3_Imdc-MOF and Zr/H_3_Imdc/PVA, including XRD, FT-IR, CHNO elemental analysis, BET surface area analysis, and SEM imaging. In addition to these shared techniques, the mechanical properties of Zr/H_3_Imdc/PVA were further evaluated by compressive strength (CS) and flexural strength (FS) measurements to assess its nanofibrous structure. Standard analytical techniques were reviewed and compared to characterize the structures of both Zr/H_3_Imdc-MOFs and Zr/H_3_Imdc/PVA.

**Fig. 2:**
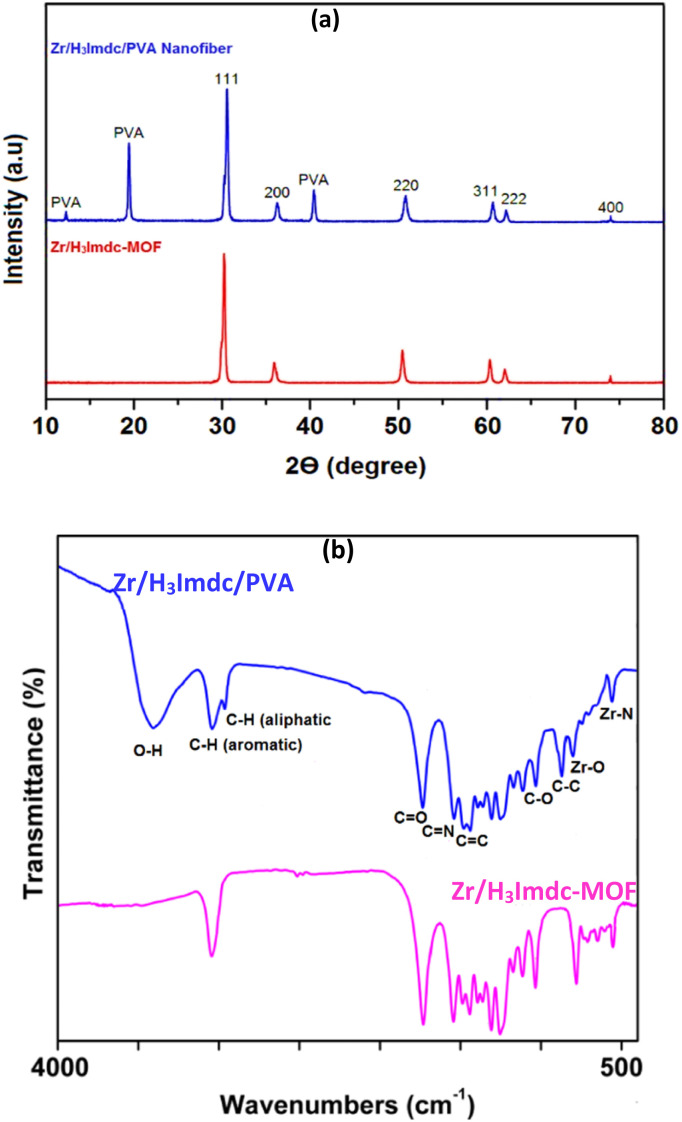
The XRD pattern (a) and FT-IR (b) spectra of Zr/H_3_Imdc-MOF and the Zr/H_3_Imdc/PVA.

The XRD pattern of the Zr/H_3_Imdc-MOF and the Zr/H_3_Imdc/PVA, as shown in Fig. 2a, exhibits similar peaks at specific 2*θ* values. These peaks, observed at 30.27° (111), 36.01° (200), 50.65° (220), 60.46° (311), 62.14° (222), and 74.09° (400), confirm the crystalline cubic structure of zirconia with JCPDS 49-1642.^54^ In the XRD pattern of the Zr/H_3_Imdc/PVA, in addition to the typical peaks, two additional peaks are present in the regions of 12.4°, 19.8° and 40.6°. These peaks are likely related to PVA based on previous studies.^55,56^

The sharp and well-defined diffraction peaks observed in the XRD patterns indicate a highly crystalline structure of the synthesized Zr-MOF. The preservation of these peaks in the composite confirms that incorporation into the PVA matrix does not disrupt the crystalline framework. Therefore, the high crystallinity indicated by the XRD patterns is a clear indicator of an effective synthesis method for these products.

The FT-IR spectra of the Zr/H_3_Imdc-MOF and the Zr/H_3_Imdc/PVA (Fig. 2b), except for the absorption bands at 3290 cm^−1^, 2850 cm^−1^, and 830 cm^−1^, exhibited nearly identical peaks in the remaining regions. The aromatic C–H peak was observed in 3010 cm^−1^, the C=O peak in 1670 cm^−1^, the C=N peak in 1540 cm^−1^, the C=C peak in 1460 cm^−1^, and the C–O peak in 1070 cm^−1^, along with zirconium–nitrogen and zirconium–oxygen peaks in 550 cm^−1^,^57^ and 790 cm^−1^,^58^ respectively.

The main index peaks observed in the spectrum of the Zr/H_3_Imdc/PVA but absent in the Zr/H_3_Imdc-MOF appeared at 3390 cm^−1^ (O–H), 2910 cm^−1^ (aliphatic C–H), and 830 cm^−1^ (C–C), respectively.

FT-IR spectra reveal noticeable changes in the intensity and broadening of the O–H stretching region after incorporation of the MOF into the PVA matrix. The observed red shift (from 3400 cm^−1^ to 3290 cm^−1^), broadening, and increased intensity of the O–H stretching band in the composite FT-IR spectrum are consistent with the formation of intermolecular hydrogen bonding between the –OH groups of PVA and the oxygen-containing functional groups (*e.g.*, carboxylate groups) of the MOF structure. These interactions likely occur between the carboxyl groups of the MOF linker and the hydroxyl groups of PVA, supporting the proposed interaction mechanism illustrated in Scheme 1b.

In addition, the absorption bands at 550 cm^−1^ and 790 cm^−1^ correspond to Zr–N and Zr–O coordination bonds, respectively, which are consistent with the proposed structures illustrated in Scheme 1.

Table 1 shows differences in carbon, hydrogen, nitrogen, and oxygen percentages between the CHNO EA results for Zr/H_3_Imdc-MOF and Zr/H_3_Imdc/PVA. The percentages of carbon, hydrogen, and oxygen in the Zr/H_3_Imdc/PVA are higher than those in the Zr/H_3_Imdc-MOF, indicating a more tremendous amount of these elements in the structure of the Zr/H_3_Imdc/PVA. Thus, both the binding of Zr/H_3_Imdc-MOF to PVA and the proposed structure in Scheme 1b for Zr/H_3_Imdc/PVA are confirmed.

**Table 1:** The CHNO EA of Zr/H_3_Imdc-MOF and the Zr/H_3_Imdc/PVA

Elements	Compounds	
	Zr/H_3_Imdc-MOF	Zr/H_3_Imdc/PVA
Percentages of carbon	25.42	42.73
Percentages of hydrogen	3.85	7.35
Percentages of nitrogen	5.36	2.94
Percentages of oxygen	13.47	20.07

Based on the proposed structures in Scheme 1, zirconium, oxygen, nitrogen, and carbon are present in Zr/H_3_Imdc-MOF and the Zr/H_3_Imdc/PVA.

High specific surface area is considered one of the most important properties of nanostructures.^59^ This property increases the potential applications of nanoparticles tailored to meet specific compound requirements.^60^ For instance, if a conventional compound exhibits adsorption properties, its nanostructured counterpart will demonstrate even greater adsorption capabilities due to its higher specific surface area, which increases the contact area with the adsorbed material.^61^ In addition, a high specific surface area greatly enhances the antimicrobial properties of nanoparticles by increasing the contact between the active ingredient of the compound and the bacterial strains under study.^62^ This property generally depends on the method used for synthesizing the nanostructure.^59,63^

**Fig. 3:**
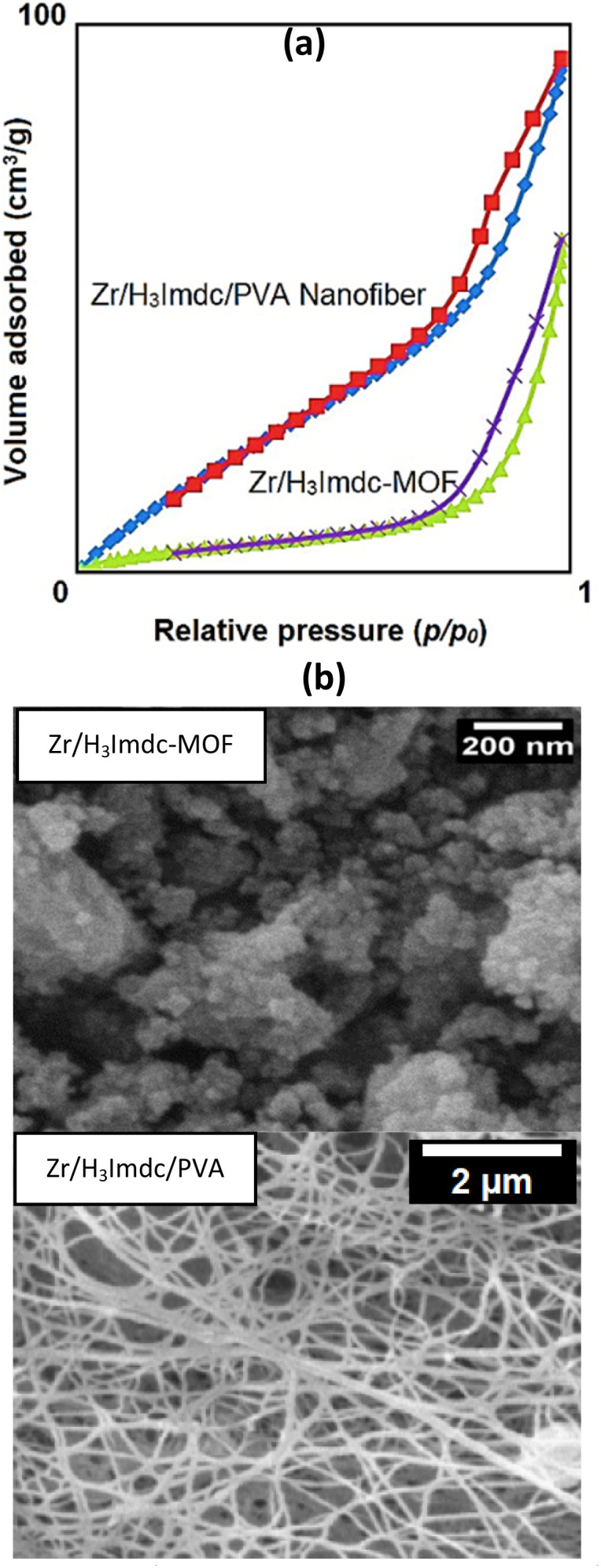
The nitrogen adsorption/desorption (a), and SEM images of Zr/H_3_Imdc-MOF and the Zr/H_3_Imdc/PVA (b).

This study revealed a high specific surface area for the synthesized products. The specific surface areas were determined using the Brunauer–Emmett–Teller (BET) method based on nitrogen adsorption–desorption isotherms measured at 77 K. The BET equation (1/[*W*((*P*_0_/*P*) − 1)] = 1/(*W*_m_*C*) + (*C* − 1)/(*W*_m_*C*) × (*P*/*P*_0_), where *W* is the weight of gas adsorbed at relative pressure *P*/*P*_0_, *W*_m_ is the monolayer capacity, and *C* is the BET constant) was applied in the relative pressure range of 0.05–0.30.^64–67^ As shown in Fig. 3a, the Zr/H_3_Imdc-MOF had a specific surface area of 1560 m^2^ g^−1^ while the Zr/H_3_Imdc/PVA had a higher value of 1755 m^2^ g^−1^.

Although detailed pore size distribution analysis was not conducted, the high BET surface area indicates a predominantly porous structure suitable for adsorption applications. Future studies will focus on pore size analysis to further clarify the contribution of micro- and mesoporosity to adsorption behavior.

The formation of multiple strong hydrogen bonds between Zr/H_3_Imdc-MOF and PVA may indirectly influence the high specific surface area of the final product by stabilizing its structure.

The obtained BET surface area of 1755 m^2^ g^−1^ is notably high for Zr-based MOF composites and exceeds many previously reported MOF/polymer systems, which typically exhibit reduced surface areas due to polymer incorporation. The high surface area observed in this study suggests that the porous framework of the Zr-MOF is largely preserved within the PVA nanofibrous matrix. This structural feature provides abundant accessible adsorption sites, which directly contributes to the high pesticide removal efficiencies observed.^6^

The final characterization of the study, which applied to both the Zr/H_3_Imdc-MOF and Zr/H_3_Imdc/PVA, involved investigating their nanosize and morphology using scanning electron microscopy (SEM) images.

The SEM micrograph of Zr/H_3_Imdc-MOF (Fig. 3b) recorded at a 200 nm scale reveals predominantly aggregated nanostructures, which can be attributed to strong interparticle interactions. In addition to these aggregates, a limited number of smaller individual features are also observable, confirming the formation of nanoscale structures.

Although SEM provides valuable information on surface morphology, the pronounced aggregation and indistinct boundaries between individual particles make precise particle size determination challenging based solely on SEM images. Such aggregation is commonly observed in MOF-based nanostructures due to high surface energy and strong interparticle interactions, including hydrogen bonding. Therefore, the nanoscale nature of Zr/H_3_Imdc-MOF and Zr/H_3_Imdc/PVA is inferred from a combination of SEM observations, their successful fabrication *via* electrospinning, and the remarkably high specific surface area obtained from BET analysis. In addition, the crystalline domains indicated by XRD patterns further support the nanostructured nature of the synthesized materials.

It should be noted that the SEM images of Zr/H_3_Imdc-MOF and Zr/H_3_Imdc/PVA were taken at different magnifications (200 nm *vs.* 2 µm) because the two materials have fundamentally different architectures.

The SC and FS are key mechanical characteristics of nanofibrous materials, and the corresponding values for Zr/H_3_Imdc/PVA were determined to be 69.1 N mm^−2^ and 17.8 N mm^−2^, respectively.

These values can be attributed to the reinforcing effect of the PVA matrix, strong interfacial interactions between Zr/H_3_Imdc-MOF and PVA, and the compact nanofibrous structure formed during the electrospinning process. The measured compressive strength (CS = 69.1 N mm^−2^) and flexural strength (FS = 17.8 N mm^−2^) indicate that the composite possesses sufficient mechanical robustness for handling and repeated use in aqueous environments. Such mechanical stability is critical for practical water treatment applications, where materials are subjected to agitation and prolonged contact with water.^3^

### 3.2 Adsorption performance

In this study, adsorption performance was primarily evaluated in terms of removal efficiency under different operational parameters. Although adsorption capacity values (*q*_e_ and *q*_max_) are commonly reported for comparative purposes, the present work focuses on removal efficiency under optimized conditions to assess the practical applicability of the composite. All adsorption experiments were performed in triplicate, and the reported values represent average results. Variations between experiments were within an acceptable range.

Previous studies have shown that one of the most important mechanisms for adsorbing compounds is the formation of bonds, particularly hydrogen bonds, between the adsorbent and the adsorbate.^68,69^ The Zr/H_3_Imdc/PVA synthesized in this study has hydrogen bonded to the oxygen on the PVA side, indicating that hydrogen bonds may also form with compounds containing oxygen or nitrogen that can participate in hydrogen bonding.

The presence of oxygen in Malathion's structure and both oxygen and nitrogen in Carbofuran's structure (Fig. 1) allows these compounds to potentially form hydrogen bonds with nanoparticles, which could enhance their interaction or adsorption.

In a study examining the adsorption properties of agricultural pesticides such as Malathion and Carbofuran by Zr/H_3_Imdc/PVA, all factors influencing the adsorption process were thoroughly examined and tested. These factors included pesticide concentration, nanofiber dosage, pH, temperature, and adsorption time.

The initial concentration of 0.02 g L^−1^ of Zr/H_3_Imdc/PVA, initial pH of 7, room temperature, and time of 60 minutes were selected as initial fixed factors.

**Fig. 4:**
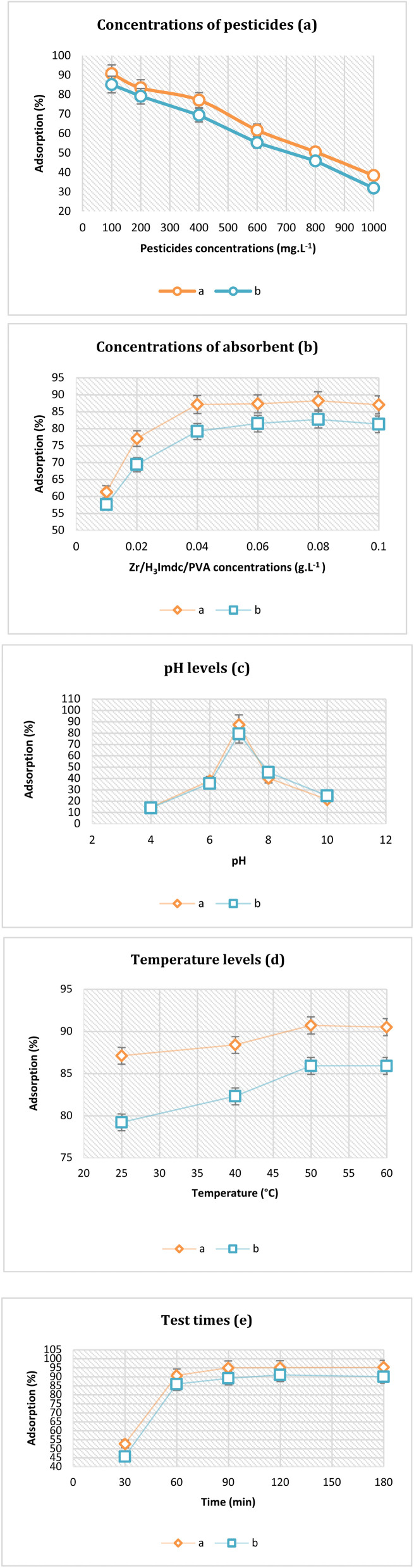
Investigation of different conditions (concentrations of pesticides (a), concentrations of absorbent (b), pH levels (c), temperature levels (d) and test times (e)) for the adsorption of pesticides using Zr/H_3_Imdc/PVA ((a): Malathion; (b): Carbofuran).

Concentrations of 100 mg L^−1^, 200 mg L^−1^, 400 mg L^−1^, 600 mg L^−1^, 800 mg L^−1^, and 1000 mg L^−1^ from pesticides were tested with a fixed amount of Zr/H_3_Imdc/PVA (0.02 g L^−1^) and it was observed that adsorption decreases with increasing pesticides concentration (Fig. 4a).

After fixing the pesticide concentration at 400 mg L^−1^, additional experiments evaluated the impact of other variables such as nanofiber dosage, pH levels, temperature conditions, and duration of exposure on the adsorption efficiency. In examining Zr/H_3_Imdc/PVA dosage (0.01 g L^−1^, 0.02 g L^−1^, 0.04 g L^−1^, 0.06 g L^−1^, 0.08 g L^−1^, and 0.1 g L^−1^), it was found that adsorption increases with increasing Zr/H_3_Imdc/PVA concentration to 0.04 g L^−1^ for Malathion and 0.04 g L^−1^ for Carbofuran, and then stabilizes (Fig. 4b).

Therefore, 0.04 g L^−1^ of Zr/H_3_Imdc/PVA was kept constant in other studies. The impact of different acidity levels ranging from pH 4 to 10 on adsorption efficiency was evaluated under uniform experimental conditions. As maximum adsorption occurred precisely at pH value around seven for both pesticides tested in our study, the highest among all tested values, subsequent analyses focused exclusively on this optimal condition (Fig. 4c).

Temperatures of 25 °C, 40 °C, 50 °C, and 60 °C were used in the study of the test temperature, and the adsorption was constant from 50 °C upwards (Fig. 4d).

The final study involved conducting the process at a temperature of 50 °C, with evaluations performed after intervals of 30 minutes, 60 minutes, 90 minutes, 120 minutes, and finally after 180 minutes. Finally, it was determined that the highest adsorption is carried out at 90 min for Malathion and 120 min for Carbofuran, and at times higher than that, the adsorption rate is almost constant (Fig. 4e).

In general, the maximum removal efficiency values was 94.9% for Malathion and 91% for Carbofuran and the corresponding optimal conditions was adsorbent dosage 0.04 g L^−1^, pH 7, temperature 50 °C, contact times 90 and 120 min.

**Scheme 2:**
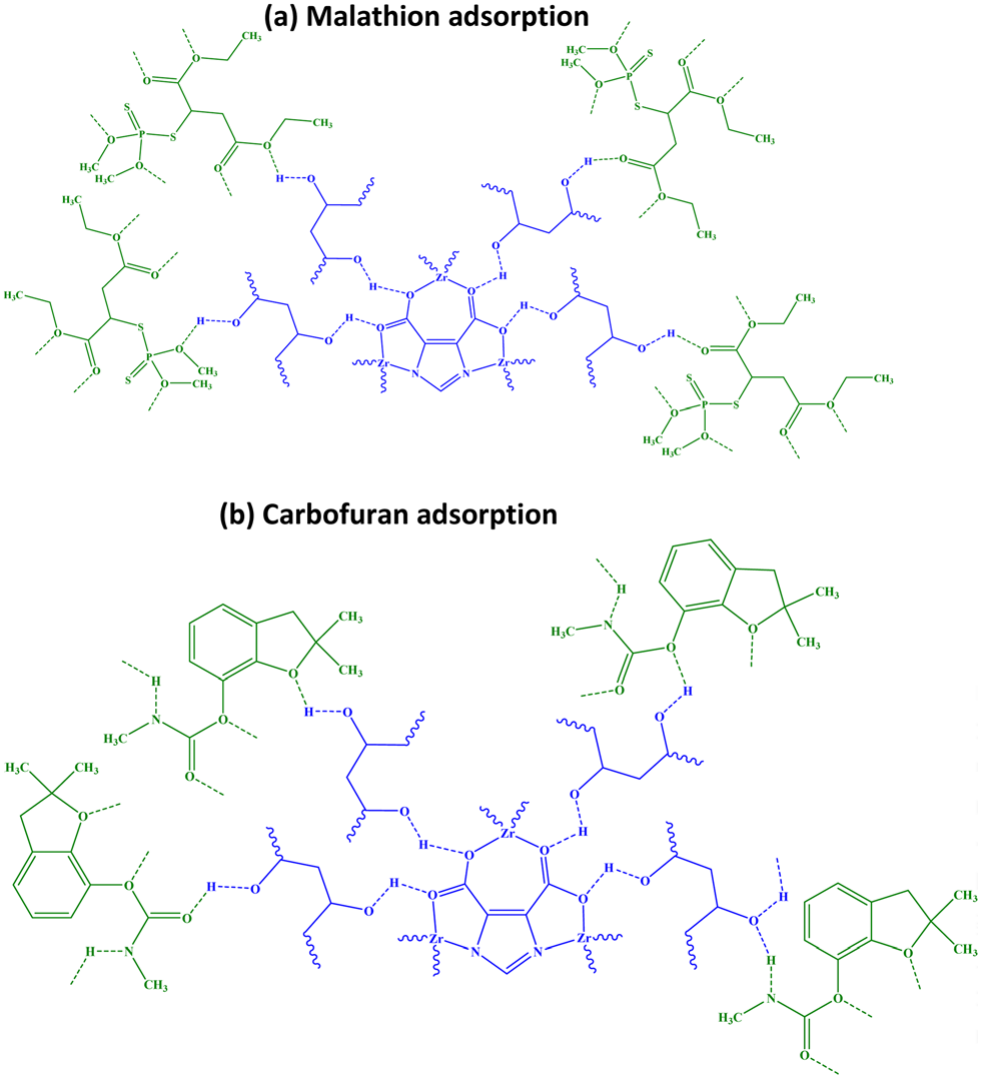
Proposed structures for pesticide ((a): Malathion; (b): Carbofuran) adsorption by Zr/H_3_Imdc/PVA.

For the adsorption of Malathion and Carbofuran by Zr/H_3_Imdc/PVA, the structures shown in Scheme 2 (a: Malathion; b: Carbofuran) are proposed, considering the active sites in their structure for hydrogen bonding. The proposed adsorption mechanism is based on plausible interactions between the functional groups of the composite and pesticide molecules. Hydrogen bonding, electrostatic interactions, and coordination with Zr-based sites may collectively contribute to adsorption. However, these interactions are proposed based on structural considerations rather than direct spectroscopic evidence after adsorption.

As observed, under fixed adsorbent dosage conditions, increasing the initial concentration of the adsorbate leads to a decrease in removal efficiency. At higher initial pesticide concentrations, the fixed number of available adsorption sites becomes saturated, resulting in a decrease in removal percentage despite continued adsorption. This behavior is commonly observed in batch adsorption systems.^70^

In the study of the amount of Zr/H_3_Imdc/PVA, it was observed that the absorption increases to 0.04 g L^−1^ of Zr/H_3_Imdc/PVA and then remains constant. At higher adsorbent dosages, a slight decrease in removal efficiency was observed, which may be associated with reduced effective surface area due to particle–particle interactions or limited mass transfer.^71^

The observed variation in adsorption efficiency with pH can be attributed to changes in the surface charge of the adsorbent and the ionization state of pesticide molecules. Under strongly acidic or alkaline conditions, protonation or deprotonation of functional groups may reduce favorable interactions, leading to a moderate decrease in removal efficiency rather than chemical degradation of the pesticides. For Malathion (which contains P=S and ester groups), deprotonation occurs above pH 7, reducing hydrogen bonding with the adsorbent. For Carbofuran (which contains a carbamate group and a furan ring), protonation of the nitrogen atom occurs below pH 5, altering its charge state and reducing interaction with the MOF surface.^72^

An increase in adsorption efficiency with temperature suggests that higher thermal energy enhances molecular diffusion and interaction between pesticide molecules and adsorption sites.^73^ Thermodynamic parameters are frequently employed to evaluate the nature of adsorption processes.^74^ However, detailed thermodynamic parameters were not calculated and will be addressed in future studies.

It was observed that the adsorption of Malathion increased up to 90 minutes and that of Carbofuran up to 120 minutes. Beyond these times, the percentage of adsorption remained relatively constant. The difference in equilibrium time between Malathion and Carbofuran may be related to differences in molecular size, structural rigidity, and diffusion behavior within the nanofibrous matrix. These factors can influence mass transfer rates independently of the number of potential interaction sites.

Therefore, 0.04 g L^−1^ of Zr/H_3_Imdc/PVA could absorb 400 mg L^−1^ of Malathion at pH 7, a temperature of 50 °C for 90 minutes. The maximum adsorption under these conditions was 94.9%. In the adsorption of Carbofuran, it was also determined that 0.04 g L^−1^ of Zr/H_3_Imdc/PVA could absorb 400 mg L^−1^ of Carbofuran at pH 7, a temperature of 50 °C for 120 minutes. The maximum adsorption under these conditions was 91%.

Comparing the structures of adsorption of Malathion (Scheme 2a) and Carbofuran (Scheme 2b), it can be seen that Malathion contains six oxygen atoms. In comparison, while Carbofuran has three oxygen atoms and one nitrogen atom. This indicates that Malathion has more sites for hydrogen bonding, which may explain its higher adsorption by Zr/H_3_Imdc/PVA.

Compared to previously reported MOF-based and polymer-supported adsorbents, the Zr/H_3_Imdc/PVA composite demonstrates competitive removal efficiency at relatively low adsorbent dosage and moderate contact time, highlighting its potential for practical water treatment applications.^75^

To better contextualize our results, Table 2 compares the adsorption performance of the Zr/H_3_Imdc/PVA composite with selected MOF-based and polymer-supported adsorbents reported in the literature.

**Table 2:** Comparison of adsorption performance of Zr/H_3_Imdc/PVA with previously reported MOF-based and nanofiber adsorbents for pesticide removal

Adsorbent	Pesticide	Removal (%)	Adsorbent dosage (g L^−1^)	BET (m^2^ g^−1^)	References
Zr/H_3_Imdc/PVA	Malathion	94.9	0.04	1755	This study
Zr/H_3_Imdc/PVA	Carbofuran	91.0	0.04	1755	This study
MOF-808	Malathion	82	0.2	1200	76
UiO-66	Carbofuran	75	0.5	1050	38
PVA/CNT nanofiber	Malathion	70	0.1	450	77
MOF/PVA composite	Carbofuran	68	0.3	890	43

Our composite achieves higher removal efficiencies (94.9% for Malathion and 91% for Carbofuran) at a significantly lower adsorbent dosage (0.04 g L^−1^) compared to most previously reported systems (typically 0.1–0.5 g L^−1^). The high BET surface area (1755 m^2^ g^−1^) of our composite also exceeds that of many conventional MOF/polymer systems, which often suffer from surface area reduction upon polymer incorporation. These comparisons highlight the potential of our Zr/H_3_Imdc/PVA nanofiber composite for efficient pesticide removal.

### 3.3 Antibacterial activity

Since one of the main objectives of this study was the removal of harmful contaminants affecting aquatic animals, the antimicrobial activity of the synthesized Zr/H_3_Imdc/PVA was evaluated against pathogenic bacterial strains common to both aquatic organisms and humans.

The selected bacterial strains represent common aquatic and fish-associated pathogens that pose significant risks in water systems and aquaculture environments. Their inclusion enables evaluation of the antimicrobial performance of the composite against microorganisms relevant to environmental water contamination rather than clinical pathogens.

This evaluation was motivated by the high specific surface area of the composite and the known antimicrobial properties of zirconium and imidazole-based components reported in previous studies.^78,79^ The results are summarized in Table 3.

**Table 3:** Antimicrobial activity of Zr/H_3_Imdc/PVA against specialized pathogenic strains common to aquatic and humans (*n* = 3 ± SD)^a^

Strains	Compounds/Parameters								
	Zr/H_3_Imdc/PVA			Cfz			Amp		
	IZD	MIC	MBC	IZD	MIC	MBC	IZD	MIC	MBC
*Edwardsiella tarda*	18.75 ± 0.9	64 ± 0.0	128 ± 0.0	—	—	—	—	—	—
*Yersinia ruckeri*	12.37 ± 0.6	256 ± 0.0	512 ± 0.0	—	—	—	—	—	—
*Streptococcus iniae*	21.08 ± 0.0	2 ± 0.0	4 ± 0.0	20.73 ± 0.5	4 ± 0.0	8 ± 0.0	22.46 ± 1.4	1 ± 0.0	2 ± 0.0
*Vibrio fluvialis*	14.31 ± 0.7	128 ± 0.0	256 ± 0.0	—	—	—	—	—	—
*Loctococcus garvieae*	17.92 ± 1.3	8 ± 0.0	16 ± 0.0	—	—	—	19.51 ± 1.8	2 ± 0.0	4 ± 0.0

a Cfz: cefazolin; Amp: ampicillin; IZD value: mm; MIC and MBC value: µg mL^−1^.

The specialized pathogenic strains common to aquatic and humans included *Edwardsiella tarda* (ATCC 15947), *Yersinia ruckeri* (ATCC 29473), *Streptococcus iniae* (ATCC 29178), *Vibrio fluvialis* (ATCC 33809), and *Lactococcus garvieae* (ATCC 43921). As shown in the Table 3, the final product demonstrated significant efficacy against these strains.

The antibacterial activity of Zr/H_3_Imdc/PVA was evaluated using inhibition zone diameter (IZD), minimum inhibitory concentration (MIC), and minimum bactericidal concentration (MBC) assays. IZD reflects the ability of the material to inhibit bacterial growth on solid media, while MIC represents the lowest concentration required to inhibit visible bacterial growth. MBC corresponds to the minimum concentration needed to completely kill the bacterial cells. Generally, when the MBC value is close to the MIC value, the antibacterial agent is considered bactericidal, whereas a much higher MBC compared to MIC indicates a bacteriostatic effect.

Bactericidal agents kill bacteria, while bacteriostatic agents inhibit bacterial growth without necessarily killing them. The distinction is typically based on the MBC/MIC ratio, where a ratio ≤ 4 is often considered bactericidal.

In the present study, the MBC values were generally twice the corresponding MIC values for all tested strains. According to standard definitions, an MBC/MIC ratio of ≤ 4 is often considered indicative of bactericidal activity. However, given that the MBC values consistently exceeded the MIC values by a factor of two, the composite may be regarded as having predominantly bactericidal potential, although a strict bacteriostatic effect cannot be completely excluded without time-kill kinetic studies.

The inhibition zone diameters (IZD) presented in Table 2 provide additional evidence of antibacterial activity. The largest IZD was observed against *Streptococcus iniae* (21.08 mm), followed by *Edwardsiella tarda* (18.75 mm) and *Lactococcus garvieae* (17.92 mm). Moderate activity was seen against *Vibrio fluvialis* (14.31 mm), while *Yersinia ruckeri* showed the smallest inhibition zone (12.37 mm). This strain-dependent variation is consistent with the MIC and MBC results, where *Y. ruckeri* also required the highest concentrations for inhibition. The IZD values correlate reasonably well with the MIC data, further supporting the antibacterial efficacy of the Zr/H_3_Imdc/PVA composite.

The observed antibacterial activity of the composite against certain strains is comparable to, and in some cases exceeds, that of standard antibiotics such as Cefazolin and Ampicillin under the tested conditions. However, this comparison does not imply clinical superiority, as antibiotics and nanocomposite materials operate through fundamentally different mechanisms of action. The tested bacterial strains were not characterized for clinical antibiotic resistance, and therefore the results should be interpreted as a functional comparison rather than a therapeutic evaluation.

Notably, while Cefazolin and Ampicillin were inactive against strains *Edwardsiella tarda*, *Yersinia ruckeri*, and *Vibrio fluvialis*, the Zr/H_3_Imdc/PVA exhibited positive antibacterial efficacy.

The antibacterial activity of the Zr/H_3_Imdc/PVA composite exhibited a clear strain-dependent behavior, as reflected by the wide range of MIC values observed among the tested bacteria. This variation suggests that bacterial susceptibility is influenced by differences in cell wall structure, membrane composition, and metabolic activity.

The notably higher susceptibility of *Streptococcus iniae* compared to *Yersinia ruckeri* may be attributed to differences in cell envelope architecture. Gram-positive bacteria possess a thick peptidoglycan layer without an outer membrane, which may facilitate stronger interactions with metal-containing and surface-active materials. In contrast, Gram-negative bacteria such as *Y. ruckeri* have an additional outer membrane that can act as a permeability barrier, reducing the effectiveness of nanostructured antimicrobial agents.^80^

The presence of imidazole and zirconium, both known for their antimicrobial properties,^78,79^ along with a high specific surface area,^62^ can be considered effective factors contributing to the antibacterial activity of the final product. Although the precise antibacterial mechanism was not experimentally investigated, the antibacterial activity of the composite is likely governed by multiple combined mechanisms rather than a single dominant pathway. The antibacterial activity can be attributed to several synergistic factors. First, the presence of zirconium species may disrupt bacterial cell membranes through electrostatic interactions and metal-ion release, leading to increased membrane permeability and leakage of cellular contents. Second, the imidazole functional groups in the MOF linker are known to interfere with bacterial enzyme systems and DNA function, possibly by chelating metal ions essential for bacterial metabolism. Third, the high specific surface area of the nanofibrous structure enhances physical contact between the active sites and bacterial cells, increasing the likelihood of membrane damage. Finally, the nanofibrous PVA matrix may facilitate controlled release of active species and improve the dispersion of MOF particles, maximizing their antimicrobial efficiency. These mechanisms are proposed based on structural considerations and previous reports, although they were not directly investigated in this study.^81^

While the antimicrobial properties of MOF-integrated electrospun nanofibers have been investigated in recent years, the focus of these studies has been predominantly on standard Gram-positive and Gram-negative model strains. Jang *et al.* (2021)^82^ demonstrated antibacterial activity of MOF/Cu_2_O nanofibers, but did not specify the bacterial strains used. Similarly, studies on MOF-based electrospun wound dressings have confirmed activity against common pathogens without detailed strain identification. Weng *et al.* (2023)^83^ developed dual-responsive nanofibers for day/night antibacterial protection, yet their testing was limited to standard laboratory strains.

More broadly, research on nano-engineered antimicrobial materials for aquaculture applications has been reported. For instance, Mosallaei *et al.* (2025)^84^ incorporated CuO nanoparticles into polyamide fish cage nets and tested antimicrobial activity, but only against *S. aureus* and *E. coli*, neither of which are primary aquaculture pathogens.

Importantly, a comprehensive search of the literature reveals that no previous study has specifically evaluated the antimicrobial activity of MOF-based electrospun nanofibers against the key aquatic pathogens *Edwardsiella tarda*, *Yersinia ruckeri*, *Streptococcus iniae*, *Vibrio fluvialis*, and *Lactococcus garvieae*. To the best of our knowledge, this is the first report demonstrating the antibacterial efficacy of Fe-MOF/PAN nanofibers against this panel of fish pathogens, thereby addressing a critical gap in the application of MOF-based nanomaterials for aquaculture health management.

### 3.4 Limitations and future directions

(1) Kinetic and isotherm modeling:

The primary objective of this work was to identify optimal operational conditions rather than to perform detailed kinetic and isotherm modeling or adsorption capacity calculations. Therefore, classical adsorption models (including pseudo-first-order, pseudo-second-order, Langmuir, and Freundlich) were not applied in this study. However, based on the optimal conditions identified here, these kinetic and isotherm studies are planned as the immediate next step of this research.

(2) Regeneration and reusability:

Regeneration and reusability studies were not investigated in the present work.

(3) Performance in complex water matrices:

Adsorption performance in complex water matrices was not evaluated.

(4) Separate contribution of MOF and PVA components:

The adsorption and antimicrobial performance of the MOF and PVA components were not tested separately. Future work should evaluate each component individually to quantify their respective contributions to the composite's overall activity.

(5) Positive control experiments with other pesticides and metals: future studies should include positive control experiments using other structurally similar pesticides (*e.g.*, diazinon or chlorpyrifos for Malathion, and aldicarb for Carbofuran) as well as composites containing other d-block metals (such as Cu or Fe) to confirm the reproducibility and generalizability of the results.

(6) Time-dependent killing kinetics and concentration-response:

Time-dependent killing kinetics and detailed concentration–response relationships were not examined. The antimicrobial assessment was limited to MIC, MBC, and inhibition zone measurements as an initial evaluation.

(7) Cytotoxicity and biosafety:

Cytotoxicity toward mammalian or non-target aquatic cells was not evaluated. Further studies are required to assess the selectivity and biosafety of the composite before practical or biomedical applications.

(8) Simultaneous performance of adsorption and antimicrobial functions:

The present study evaluated adsorption and antimicrobial functions independently. Future studies will focus on assessing the simultaneous performance of both functions under realistic conditions.

(9) Long-term stability and economic feasibility:

Long-term stability and economic feasibility were not addressed in this work.

(10) Mechanistic studies:

Mechanistic studies using spectroscopic and computational methods are recommended to optimize adsorption and antimicrobial pathways, enabling pilot-scale application in aquaculture and contaminated water systems.

## 4 Conclusions

This study successfully developed a bifunctional Zr/H_3_Imdc/PVA nanofiber composite capable of simultaneous removal of pesticides (Malathion and Carbofuran) and control of aquatic bacterial pathogens. The composite exhibited exceptional adsorption performance, achieving 94.9% removal for Malathion and 91% for Carbofuran with only 0.04 g L^−1^ adsorbent, significantly lower than typical MOF-based dosages (0.1–1.0 g L^−1^). Optimal conditions (pH 7, 50 °C, contact times 90–120 min) are practical for water treatment applications. High surface area and hydrogen-bonding capability facilitated efficient pesticide capture. The composite also demonstrated potent antimicrobial activity against five aquatic pathogens, including strains resistant to β-lactam antibiotics (MIC: 2–256 µg mL^−1^). The combined effects of Zr^4+^ ions, imidazole groups, and nanofiber architecture contributed to broad-spectrum activity. Mechanical testing (CS = 69.1 N mm^−2^, FS = 17.8 N mm^−2^) confirmed the material's structural integrity.

## Author contributions

F. M. A. A.: writing – original draft, and writing – review and editing, formal analysis; M. A. H.: writing – review and editing, investigation; S. M. D. Y.: writing – original draft, conceptualization; K. H. A.: writing – original draft, methodology; W. M. T.: writing – review and editing, resources; A. A. A.: writing – review and editing, visualization; M. J.: writing – review and editing, validation; H. M.: writing – original draft, data curation; I. A.: writing – original draft, writing – review and editing, data curation; A. S.: writing – review and editing, software. All authors have read and approved the final version of the manuscript.

## Conflicts of interest

There are no conflicts to declare.

## Data Availability

It is confirmed by the authors that the data necessary to support the findings of this study can be found in the article.
